# Using archaeogenomic and computational approaches to unravel the history of local adaptation in crops

**DOI:** 10.1098/rstb.2013.0377

**Published:** 2015-01-19

**Authors:** Robin G. Allaby, Rafal Gutaker, Andrew C. Clarke, Neil Pearson, Roselyn Ware, Sarah A. Palmer, James L. Kitchen, Oliver Smith

**Affiliations:** 1 School of Life Sciences, University of Warwick, Gibbet Hill Campus, Coventry CV4 7AL, UK; 2 Rothamsted Research Station, Harpenden, Hertfordshire AL5 2JQ, UK

**Keywords:** ancient DNA, domestication, local adaptation, archaeogenomics

## Abstract

Our understanding of the evolution of domestication has changed radically in the past 10 years, from a relatively simplistic rapid origin scenario to a protracted complex process in which plants adapted to the human environment. The adaptation of plants continued as the human environment changed with the expansion of agriculture from its centres of origin. Using archaeogenomics and computational models, we can observe genome evolution directly and understand how plants adapted to the human environment and the regional conditions to which agriculture expanded. We have applied various archaeogenomics approaches as exemplars to study local adaptation of barley to drought resistance at Qasr Ibrim, Egypt. We show the utility of DNA capture, ancient RNA, methylation patterns and DNA from charred remains of archaeobotanical samples from low latitudes where preservation conditions restrict ancient DNA research to within a Holocene timescale. The genomic level of analyses that is now possible, and the complexity of the evolutionary process of local adaptation means that plant studies are set to move to the genome level, and account for the interaction of genes under selection in systems-level approaches. This way we can understand how plants adapted during the expansion of agriculture across many latitudes with rapidity.

## 1. Introduction

During the closing phases of the last glacial stage that had predominated the climate system for previous 100 000 years or so, a number of plant species became adapted to an emergent human environment independently at different centres around the globe. This process led to the evolution of domesticated and commensal species. Initially, the evolution of domestication involved the selection of a characteristic group of traits collectively termed the domestication syndrome [1,2]. These traits, which included the loss of shattering, changes in seed size, loss of photoperiod sensitivity and changes in plant and floral architecture [3], enabled the better survival of plants in the human environment. That this was an adaptation to the human environment by plants is emphasized by the fact that a number of non-food plants such as small-seeded grasses and legumes also adapted to this environment under the same regime of cultivation and became commensals, and indeed also display traits of the domestication syndrome [4–6].

Evolution is an interminable process, and the story of the evolution of domestication did not end with the emergence of those first adaptors to the human environment. Some of the commensals later went on to become domesticated crops themselves, such as in the case of oats and rye (reviewed in reference [7]). The human environment to which the plants had adapted was dynamic and presented plants with new challenges, resulting in new adaptations and also new winners and losers among the domesticated species [8]. One of the greatest challenges was undoubtedly the spread of agriculture from various centres of origin to new latitudes, in almost all cases far away from the biogeographic distribution of the wild progenitor species. For plants, the environment changed in terms of temperature, rainfall and daylength on a grand scale, particularly for instance as crops were dispersed northwards into Europe. Further demands were placed on plants on a local and regional scale as they were moved to new soil types and specific environmental conditions, such as high altitude or arid environments. Alongside this, cultural innovation and changing agrarian practices altered the selection regime [9]. Evidence is emerging of the adaptation of different cultural complexes to specific ecological niches as agriculture spread into Europe [10], and stalls occurred in the spread of agriculture that are associated with a combination of the time required for the adaptation of crops to new environments as well as to the changing assemblage composition of the agrarian package itself [11,12]. In both Europe and Asia, the push northwards was associated with adaptations to changing daylength [13–15], and with commensals better adapted to the northern ecologies, such as European rye making the transition from commensal to domesticated species [16]. There is no definable endpoint to the evolution of domestication, and it is a process that should be considered as ongoing [17].

## 2. Crop adaptation to complex environments

### (a) Ancient DNA as an approach to studying local adaptation

**Figure 1.**
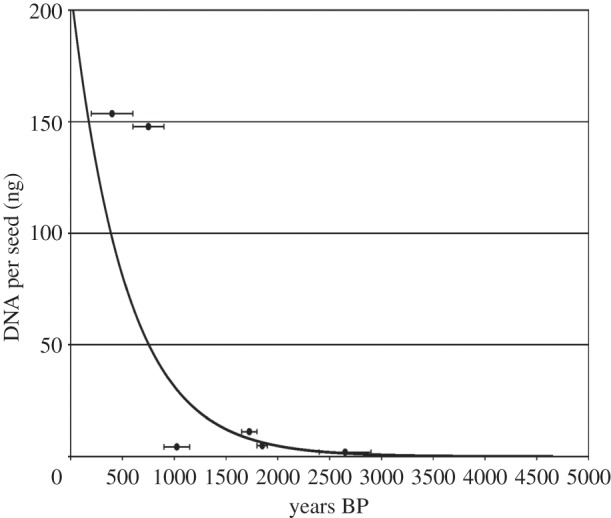
Graph shows DNA content of desiccated barley seeds over time in Qasr Ibrim, North Africa [19].

The challenges facing plants and humans were complex and dynamic. How and to what extent plants could adapt to complex environments and how much change could be embraced within their sphere of plasticity are questions of importance to understanding evolution in general, as well as the emergence and spread of agriculture. Ancient DNA (aDNA) provides an inroad to understanding that evolutionary process directly. Although the potential of aDNA in understanding the spread of agriculture was recognized in the 1990s [18], major obstacles became apparent. One major obstacle was that preservation of DNA was largely unsuitable for large-scale analyses with the technology of the time. There are two aspects to this obstacle. The first is that it is an inherent problem that the rise of agriculture took place in locales of low latitude where relatively warm temperatures limit to just a few thousand years the time depth from which ancient biomolecules can be retrieved (figure 1) [19]. The second is that the vast majority of archaeobotanical material are in the form of charred remains and a few species mainly found under waterlogged conditions. In the case of charring, evidence of DNA preservation was found to be at best sporadic [20]. Partly owing to these limitations, the number of studies of the evolution of domestication of plants that used aDNA was low relative to other research areas in aDNA [21–23]. Despite these limitations, some glimpses into the evolution of crops have been possible. An extinct expansion of a wheat crop type was detected using charred material that could have reflected an ecological limit and failure to adapt to the dynamic human environment [24]. However, insights using charred material were restricted to the sporadic establishment of the phylogeographic presence or absence of small markers from which little could be inferred about how evolution or local adaptation had occurred [25–27]. The potential to observe selection and adaptation to the human environment directly through aDNA was first achieved with desiccated remains of maize, in which three biologically significant genes were surveyed over time and space in a handful of samples [28]. While these incremental advances using aDNA offered tantalizing glimpses of the evolutionary process, the relatively small datasets generated meant that progress was prohibitively slow until the advent of next-generation sequencing a few years later.

### (b) Genetic expectations revealed through models

A second major obstacle to understanding how plants adapted to complex environments was a wider problem of accurate interpretation of genetic diversity that has been produced by complex processes [29–32]. This problem became apparent when interpretations of genetic data became increasingly divergent from the evidence unearthed in archaeology [30]. On the one hand, a long-held assumption of the high strength of artificial selection giving rise to a rapid and geographically definable origin of crop domestication was supported by many genome-wide-based analyses. On the other hand, archaeological evidence suggested a long protracted arrival of domesticated forms of cereal crops, with a hitherto unappreciated long period of pre-domestication cultivation that stretched thousands of years back into the Pleistocene [33,34], and a slow subsequent fixation of traits over a period of thousands of years [35].

Increasingly, computational models are being applied to phylogeographic data to assess alternative domestication history hypotheses. Modelling has revealed that the genetic inferences were based on analysis of data with low discriminatory power, and in fact, genetic data diversity is compatible with the notion of a protracted origin [30]. More precise estimates of the strength of domestication syndrome traits directly from the archaeological record have led to the further unexpected conclusion that the selection coefficients involved are low (in the order of 0.003) for traits as divergent as shattering, largely under monogenic control and increased seed size, under polygenic control [36]. This level of selection is more akin to natural selection than the popular perception of artificial selection. These results are surprising given that field experiments have shown that selection under cultivation can be strong [37]. Consequently, it has now become a central question to understand how plants became adapted to the human environment, and why it took as long as it did. This poses a second question: how much selection could have occurred? Haldane [38] first formally recognized that one could not have unbridled amounts of selection, because selection necessarily comes at a cost. In order for differential survival to occur, some individuals have to die (or fail to be born). For this reason, Haldane concluded that plant breeders are limited in the number of traits they can breed into varieties. A model of the number of genes, intensity of selection and probability of population survival shows that the limit to the number of genes that could be under selection is in the order of 50–100 for plant populations at the levels of intensity observed in the archaeological record [39] (electronic supplementary material, figure S1*a*). Interestingly, these values are similar to the number of genes showing signatures of selection from genome studies of crops such as maize, wheat and sunflower in which estimates vary from 27 to 70 genes under selection [40–43]. Another important insight from this model comes from the total amount of selection (the selection load) that occurs under different selection intensities per locus (electronic supplementary material, figure S1*b*). Here, we find that more selection can occur at lower selection intensities. An interpretation of this is that a greater amount of selection can occur under complexity than can occur under strong selection. Furthermore, a selective sweep may render a population vulnerable to further change, making it less able to cope with a dynamic human environment and restricted to effective adaptation within low complexity environments. To put it another way, we expect from these models for complex selection to be more robust than selective sweeps. This analysis begs the question about the nature of the human dynamic environment to which plants have adapted—can it be considered complex or simple? It may be tempting to speculate that cultivation should be considered a simple environment in which humans negate many of the issues plants would have to cope with in the wild, but the evidence from the archaeological record suggests otherwise [11].

The models outlined here suggest to us that we should expect a number of loci in the order of 50–100 under relatively weak selection. A corollary of this mode of change is that it seems likely that traits would be targeted at multiple loci weakly to effect strong selection rather than the more conventional perspective of a strong selective sweep at a single locus. It is therefore a prediction that we should see multiple changes in the interactions of genes and their products, such as regulatory and metabolic networks.

## 3. Complex adaptations viewed through ancient DNA and next-generation sequencing

### (a) Large amounts of genome evolution over short time periods

The advent of next-generation sequencing (NGS) opened up the real possibility of using aDNA to track evolution directly and test the expectations of genetic diversity generated through models and the archaeological record. One approach to look at large-scale genome evolution is to monitor the change in the transposable element (TE) composition. Cotton provides a good example of a crop in which to study the evolution of genome architecture in this way, because evidence from interspecific comparisons suggests that there have been recent significant expansions and contractions of TEs [44–46]. We were astounded to observe the extent of change of retroelement composition in the diploid species *Gossypium herbaceum* (electronic supplementary material, figure S2). In this case, 454 Roche FLX shotgun metagenomic data were generated from four samples of desiccated archaeological cotton from Africa, Brazil and Peru, and compared with data from modern accessions [47]. *G. herbaceum* is thought to be a very young species, speculated to be little older than the Holocene [48]. This youth is supported by the observation that lineage sorting appears to be very incomplete between *G. herbaceum* and its sister taxon *Gossypium arboreum* in which we found out of 10 PCR systems none yielded alleles that were exclusive to one or other species in a sample of 91 accessions (SA Palmer, AJ Clapham, P Rose, F Freitas, BD Owen, D Beresford-Jones, JD Moore, JL Kitchen, RG Allaby 2012, unpublished data). We therefore find it surprising that such differentiation in TE proportions is observed within *G. herbaceum*. This finding contrasted with the tetraploids (*G. hirsutum* and *G. barbadense*) in which we found very little change within and between species. The tetraploid species are related to the diploid species through a genome donation of an ancestor of the diploids around 1.5 Ma [49]. The contrasting pattern between the diploids and tetraploids appears to be reminiscent of punctuated equilibrium, which has recently been linked to TE composition and turnover [50,51]. In this case, more work is needed to explore cotton genome evolution directly. For example, tracing older cotton genomes would enable us to see the development of expansions over time and establish whether we see a reduction of diversity closer to the origin of speciation, or whether there is standing variation that could better explain our results that lies in stark contrast to the invariant tetraploids which were sampled from a wider spatial and temporal range.

While the evidence of TE change over time would appear to support the expectation of large amounts of small change (assuming most transpositions had little effect on the genome functionality), they tell us little about the adaptive value of such change but hint at the potential pace of change and so capability of adaptation that could be possible. In this particular study, we considered fragments of retrieved DNA that fell in gene regions as a possible source of information about adaptive change. Gene variants from these types of data may represent allelic variants or sequencing errors (which occur at a rate of about 1% for the platform used). However, we would expect sequencing errors to be randomly distributed throughout the genome, but our expectation from the models outlined above is that variants are likely to be clustered non-randomly in gene networks. We identified 210 gene fragments from our cotton metagenomic dataset that differed from public database entries and of those we were able to map 20 to the KEGG (http://www.genome.jp/kegg/) metabolic pathways map (electronic supplementary material, figures S3 and S4). It is notable that of these 20 variants, 17 fall in close proximity to another variant in their respective part of the metabolic network, on average separated by three nodes from their nearest neighbour. In this analysis, six metabolic clusters are apparent, supporting the notion that they are not random and appear to fall in line with the model predictions of multiple changes within gene networks. The incorporation of such approaches in the study of local adaptation holds the promise of systems level insights through aDNA.

### (b) Qasr Ibrim, Egypt: a site of local adaptation?

This early work with NGS in archaeobotanical remains established that complex genome level insights into the evolutionary process could be gained from samples of low latitude sites. The archaeological site at Qasr Ibrim, Egypt, provides an opportunity to expand on these approaches. Qasr Ibrim was a boundary settlement on the edge of the Nubian and Roman Empires located between the first and second cataracts of the Nile, and was occupied by five successive cultures: Napatan, Roman, Meroitic, Christian and Islamic [52]. The site is very dry, and the preservation of archaeobotanical remains is remarkably good [53]. There is a continuous record of occupation over 3000 years that provides an ideal opportunity to study crop evolution through time. Of particular interest is the barley found at the site that appears to be a two-row form that has evolved from a six-row form [19]. In modern barley, two-row architecture is the wild state, caused by a transcription factor *Vrs1* that inhibits the development of the two lateral florets in a flower spike. The six-row architecture is caused by a loss of function of this transcription factor allowing lateral floret development [54]. The barley at Qasr Ibrim is curious because it has the non-functional version of the transcription factor, so should be six-row form. A simple model shows that the fecundity difference incurred by the architectural change between row types means that six-row barley is expected to quickly outcompete two-row, within the lifetime of a farmer [19]. Fitting with this expectation, the six-row type emerged very early in the domestication of barley [55]. The predomination of two-rows in the wild suggests that they must have a strong selective advantage over six-row in the face of the fecundity difference, and indeed, under conditions of water stress, two-row barleys fair better [56]. We hypothesized that the Qasr Ibrim barley may represent a local adaptation to the dry conditions of the Upper Nile. If so, then it may be the case that other genes show functional changes associated with adaptations to do with water usage and drought tolerance. We have explored several approaches to using NGS to study the ancient nucleic acids of the barley of Qasr Ibrim that illustrate what is possible, and which paint quite an unexpected picture of the history of barley in this region.

### (c) Survival of and insights from ancient RNA

**Figure 2.**
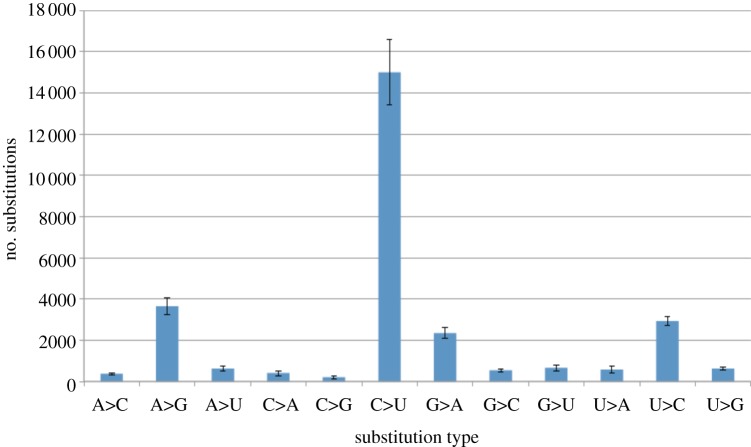
Base modification frequencies of RNA observed from archaeological barley stripe mosaic virus (BSMV) [62].

While DNA contains the evolutionary record of the genome, RNA has the potential to offer insights into the last activities of the organism through a record of gene expression. Although they were among the earliest in the field [57], few studies have been carried out on ancient RNA because it is expected to degrade about 50-fold faster than DNA, largely because it is highly prone to hydrolytic attack [58–60]. Consequently, under arid conditions, one might expect some preservation of RNA. Recently, NGS has been successfully applied to RNA from desiccated maize kernels [61]. We were surprised to find that the RNA content of the barley at Qasr Ibrim is actually higher than that of DNA [62]. At the point of death, RNA content is expected to be higher than DNA because of the multiple copies of RNA that exist relative to DNA gene copies. Assuming a ratio of between 5- and 100-fold more RNA than DNA at the point of deposition, we estimate that at Qasr Ibrim the rate of RNA decay is in the order of two- to fourfold greater than DNA, a much reduced rate than expected, most likely owing to the very arid conditions of this site and consequent reduced rate of hydrolytic attack. The diagenetic process of base modification appears to be similar in this RNA to that found in aDNA with frequent conversion of cytosine to uracil, most likely through deamination as with DNA (figure 2). There are, however, interesting differences also in the RNA degradation relative to DNA. The distribution of cytosine base modifications mapped through mapDamage v. 2.0 [63] is biased towards both ends of the molecule in RNA, rather than towards the 5′ end of reads in dsDNA where exposed overhangs are more prone to hydrolysis. We hypothesize that this may be due to secondary structures forming primarily over the central part of the molecule and sheltering it from chemical attack.

We examined the RNA portion of the barley at Qasr Ibrim using Illumina sequencing technology to see if we could learn anything about the regulatory action of microRNAs [64]. Generally, we have succeeded in recovering miRNAs from archaeological barley, and we do view differences in the relative expression profiles of archaeological and modern barleys that seem to indicate that the barley of the Christian era was stressed. Briefly, our unpublished data (RG Allaby, R Gutaker, AC Clarke, N Pearson, R Ware, SA Palmer, JL Kitchen, O Smith 2013) show the presence of miRNAs in the Christian era associated with germination inhibition, suggesting the avoidance of growth under harsh environmental conditions. An unexpected result was the retrieval of the first RNA genome that belonged to the barley stripe mosaic virus (BSMV) [62]. Historical records attributed to this virus only go back for the past 100 years or so, and analysis of modern genomes suggests an age of origin no greater than 200 years. The inclusion of the ancient RNA genome indicates that the Qasr Ibrim virus is close to the base of the crown group, suggesting an expansion of this virus contemporaneous with the Crusades, with an origin some time before this. BSMV has no known vector and is spread by physical contact between grains or pollen [65], so its rise at this time may have been linked to the intensification of agriculture that occurred to support the medieval war machine.

The RNA results demonstrate that it is possible to identify activated points of gene networks directly from the past. Furthermore, viruses play an important role in the adaptation of organisms to new environments [66]. Viruses resident to an indigenous community may affect newcomers more severely than their indigenous hosts, and likewise the introduction of viruses by newcomers may affect the indigenous community severely. In this respect, viruses can be considered an important part of the adaptive arsenal carried by organisms rather than simply a burden. The movement of domesticated plants throughout history and particularly at a more global level in recent times is of concern regarding the emergence of new diseases that affect our food supply [67]. Therefore, viruses add an important dimension to the understanding of the local adaptation of crops that is visible through the archaeobotanical record.

### (d) Methylation patterns

**Figure 3.**
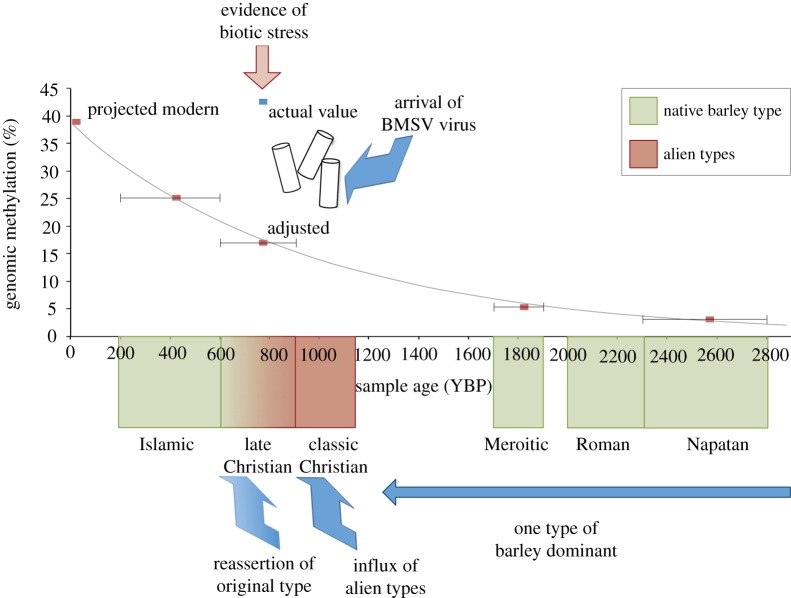
A perspective of the history of barley in Nubia. After [62,69] and RG Allaby, R Gutaker, AC Clarke, R Ware, SA Palmer, O Smith, W Nicholson, L Kistler 2013, unpublished data.

The global methylation state of a plant genome can be informative about the level of stress it is under. Methylation of cytosine bases causes the silencing of genes and is an effective genomic mechanism to control TEs. Typically, up to 90% of plant genomes can become methylated under conditions of stress [68]. Given the emergent picture of the barley at Qasr Ibrim, and our initial suspicions of water stress at the site, we were interested to know whether anything remained of the methylation signal under these preservation conditions [69]. Using the MethylMiner kit, the CpG methylation signal of archaeological barley through time was established (figure 3). The methylation signal falls exponentially over time, and extrapolation of the trend to modern times results in methylation levels which are in the normal range for barley. In ancient samples, the strength of the methylation signal is expected to be less than modern, because the shorter DNA fragments that are bead captured will contain fewer methylated cytosine sites. However, the decrease in signal, in this case, we believe is due to chemical modification of the methylation signal rather than DNA fragmentation, because the size distribution of the DNA fragments did not vary greatly between archaeobotanical samples of different ages. The barley that corresponds to the Christian strata is notable because its signal suggests 98% methylation of the living barley, indicating a high degree of stress. Therefore, the barley at Qasr Ibrim was not stressed as far as we can see, until the Christian era, and returned to normal methylation levels after that time in the Islamic era. We confirmed this pattern using bisulfite sequencing of a region of the *eIF4* locus that again showed the high degree of methylation associated with the Christian era.

### (e) The possible utility of charred grains

**Figure 4.**
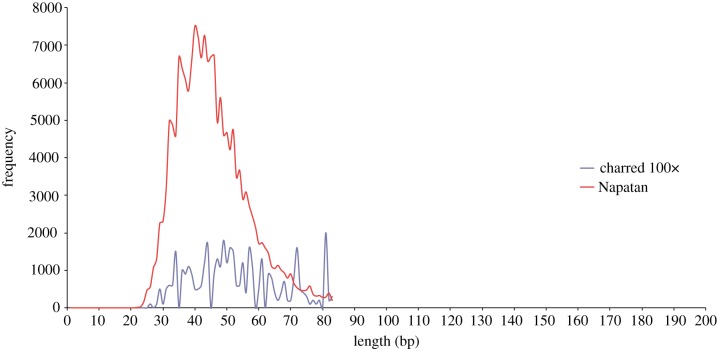
Size distribution of DNA reads from 2000 year old charred and 2500 year old desiccated barley (Napatan) obtained through DNA capture and Illumina sequencing.

A DNA capture approach was also applied to the barley of Qasr Ibrim through time. A chip of 183 genes selected for their possible roles in drought adaptation was used to capture DNA from multiple points in the strata, which was sequenced using Illumina technology (RG Allaby, R Gutaker, AC Clarke, R Ware, SA Palmer, O Smith, W Nicholson, L Kistler 2013, unpublished data). Some preliminary overviews of the results are presented here to help complete the picture of the use of NGS and aDNA to study local adaptation in barley at Qasr Ibrim. Of particular interest was a single 2000 year old (Roman) charred grain of barley from Kawa for which we obtained a few reads (amounting to 82 000 bp of reads in total). A frequency distribution of read lengths obtained from this sample, and compared with a desiccated sample of slightly greater age (Napatan) shows that the two are essentially identical in the size range and differ principally in absolute frequency (figure 4). While all interpretations should be cautious because of biases in size distribution introduced by the library preparation process, the similarity of these profiles is surprising and encouraging. Other research groups have managed to produce shotgun NGS data from charred wheat using alternative platforms [70], and sequencing technology has now reached a point at which the vast charred archaeobotanical record may be accessible to a useful degree.

### (f) The crusades as an example of the introduction of poorly adapted crops

At the time of writing, we have recovered alleles of 86 loci from 52 single grains of barley from Napatan, Roman, Meroitic, Christian and Islamic levels at Qasr Ibrim (data not shown). Contrary to our expectations, the barley at Qasr Ibrim is not very distinct from the barley of Nubia and the Near East generally. However, we do see a distinct influx of different alleles during the Christian era.

In each of the ways we have examined the Qasr Ibrim barley, we have found that the Christian era is the distinct stratum. This era is contemporaneous with the arrival of the crusaders, during which time we see distinct barley, the arrival of a virus and a methylation signature of stress. An interpretation that we are now exploring is that the crusaders may have brought barley with them that was less able to cope with local conditions than the barley of the region. The original barley type appears to have generally been reasserted during the Late Christian and completely by the Islamic phases. If the barley that was resident at Qasr Ibrim before this time was truly locally adapted to the site, then the signature of that adaptation is more subtle than the level of resolution our analyses have currently reached. This would be in keeping with expectations from the models that demonstrate that the number of loci that can be under selection is limited, and the effect of any one locus likely small. In such a model, we might not expect fixation of differences, and that different combinations of alleles may achieve a selectively similar outcome. Qasr Ibrim still has a good deal to teach us about local adaptation, and further unexpected turns may come to light.

## 4. Concluding remarks: archaeogenomes to systems

### (a) Getting behind introgressions

Technology has progressed to a level that allows the evolution of crops to be studied at a truly genomic level, and the next step is undoubtedly the retrieval of the first complete plant genomes from the archaeological record. This will enable us better to gauge the accuracy of the predictions of models for how evolution and selection have proceeded in domesticated crops. Models give us a framework in which we expect, for the most part, that selection has been weak. A corollary of this prediction is that if we sequence ancient plant genomes that come from a time closer to the onset of entering into the human environment at the beginning of the domestication trajectory then we should expect to see stronger signals of selection at loci in which we see no signal in modern genomes. Furthermore, as plants evolve along the domestication trajectory over long periods of time, and move to new environments where new wild populations are encountered, there is ample opportunity for introgressions to occur which have large functional consequences on the domesticated crop. For instance, the majority of the known domestication syndrome associated genes of Indian rice have been acquired, through introgression, from Japanese rice that arrived in the Indian subcontinent [71–76]. In this way, the domesticated crop was able to use adaptations of the different wild races to local environments. While modern genomes are the palimpsest of these complex histories and difficult to interpret, aDNA approaches will be able to unravel the sequence of acquisitions through introgression by sequencing genomes before and after such events. These approaches will help identify the origins of various parts of the genome and consequently the environments to which they had been adapted prior to introgression, as well as establishing the likely order of trait acquisition through introgression.

### (b) Low complexity adaptations in crops to complex environments?

Our models lead us to expect a large number of genes under weak selection rather than under one or two genes under strong selection, which appears to be the emergent picture from studies of adaptive evolution in wild plants [77–79]. They also lead us to expect that gene networks and metabolic processes would be affected at multiple points. Interestingly, such a pattern has recently been observed in the adaptation of dogs to a starchy diet in the human environment [80]. This indicates that an important frontier to broach in genomic-level analyses is to account for how selection acts on genes that are interdependent in networks of interaction—to move to a systems level of analysis. At a systems level, the majority of adaptation is expected to be achieved through the up- and downregulation of members of gene networks, effecting rapid and complex responses to environmental stimuli [81]. The DNA binding sites for transcription factors involved in regulation are very simple and consequently frequently form and disappear spontaneously with mutation, answering to some extent Haldane's original assertion in his contribution to the modern synthesis that the majority of mutations are expected to result in the loss of function owing to the expectations of entropy [82]. This elegant description of evolution implies the retention of function in genes during most adaptive change. However, these expectations are not met when we consider the adaptation of domesticated plants, such as wheat, barley and rice, to higher latitudes. Instead of adjusting the expression of genes in the networks of the floral pathway to attune to these new latitudes as we might expect to have occurred naturally, we see irreversible loss-of-function mutations in the associated gene networks (reviewed in reference [13]). It was this that, in many cases, helped plants to move with the human environment into northerly latitudes in which survival of the winter season would not have been assured.

These adaptations of domesticated cereals to latitude appear crude, one-way and simplistic relative to what we would expect of natural systems. In many cases, a single mutation achieves the phenotype rather than a number of mutations with each contributing a small effect. Furthermore, these genotypes probably rose in frequency relatively rapidly given the rapid pace of the spread of agriculture [11]. Could these be examples of selective sweeps that are, by corollary, rapid adaptations of low complexity? Under this scenario, it could have been the pace of agricultural movement across latitude that drove the intensity of selection, demanding rapid adaptation by plants in the human environment. Our models suggest that populations under strong selection will be vulnerable to further selection pressures that could cause population collapse, because the overall cost of selection would be too high. Intriguingly, the archaeological record indicates that such processes may have occurred—we see repeatedly that agriculture arrived at certain latitudes and collapsed not long after [83,84].

Ancient DNA and models have an important role in facilitating understanding of local adaptation in the future at a systems level. In the case of adaptation to latitude, models need to be applied to determine what the expectations are for the evolution of gene networks moving over latitude [85]. Does rapid movement across a selective gradient lead to the expectation of the retention of loss-of-function mutations that effectively break gene network interactions? Would a slower pace of movement been more likely to have led to a more refined adjustment response in networks? Archaeogenomics provides a reasonable approach to track the timing and order of the occurrence and subsequent selection of the mutations involved. In the case of studying the adaptation to latitude, aDNA technology will have to make further inroads in to using DNA from charred material. The resulting insights into how evolution works will be of relevance to understanding how plants adapted to the complex and dynamic human environment in the past, and how they might do so in the future in an ever changing world.

## Supplementary Material

Supplementary figures
