# Supplementary material for: Using archaeogenomic and computational approaches to unravel the history of local adaptation in crops
Source: Philos Trans R Soc Lond B Biol Sci. 2015 Jan 19;370(1660):20130377. doi: 10.1098/rstb.2013.0377 (PMC4275885; doi:10.1098/rstb.2013.0377)
Supplement: Supplementary figures [file rstb20130377supp1.pdf]

Using archaeogenomic and computational approaches to unravel the history of local adaptation in crops

Robin G. Allaby[1*], Rafal Gutaker[1], Andrew C. Clarke[1], Neil Pearson[1], Roselyn Ware[1], Sarah A. Palmer[1], James L. Kitchen[2] and Oliver Smith[1].

1. School of Life Sciences, Gibbet Hill Campus, University of Warwick, United Kingdom, CV4 7AL
2. Rothamsted Research Station, Harpenden, Hertfordshire, United Kingdom, AL5 2JQ

Supplementary Figures

Figure S1. A. Probability landscape of population survival (*p*) for a given number of loci (*g*) under selection coefficient (*s*) for an inbreeding cereal such as barley. B. Linear regression of the maximum selection load (total amount of selection) carried across loci for different selection coefficients without any population extinction in simulations (from Allaby, Fuller & Kitchen 2014).

Figure S2. Genomic profiles of Gypsy-, Copia-, and LINE-type retrotransposons in modern and archaeobotanical cotton. (Palmer *et al.* 2012b).

Figure S3. Cluster analysis of archaeological *Gossyium barbadense* read variants mapped to *Arabidopsis thaliana* metabolic Pathways**.** Reads were mapped to the KEGG *A. thaliana* metabolic pathway. The number of major nodes between reads were used to create a distance metric between variants, which were then grouped by

K-median clustering using the “Pheatmap” package in R. Groupings have 7 nodes or fewer between all points.

Figure S4. Archaeological *Gossyium barbadense* read variants mapped to *Arabidopsis thaliana* metabolic Pathways. Variants are represented by stars, for colour key see Figure S3.

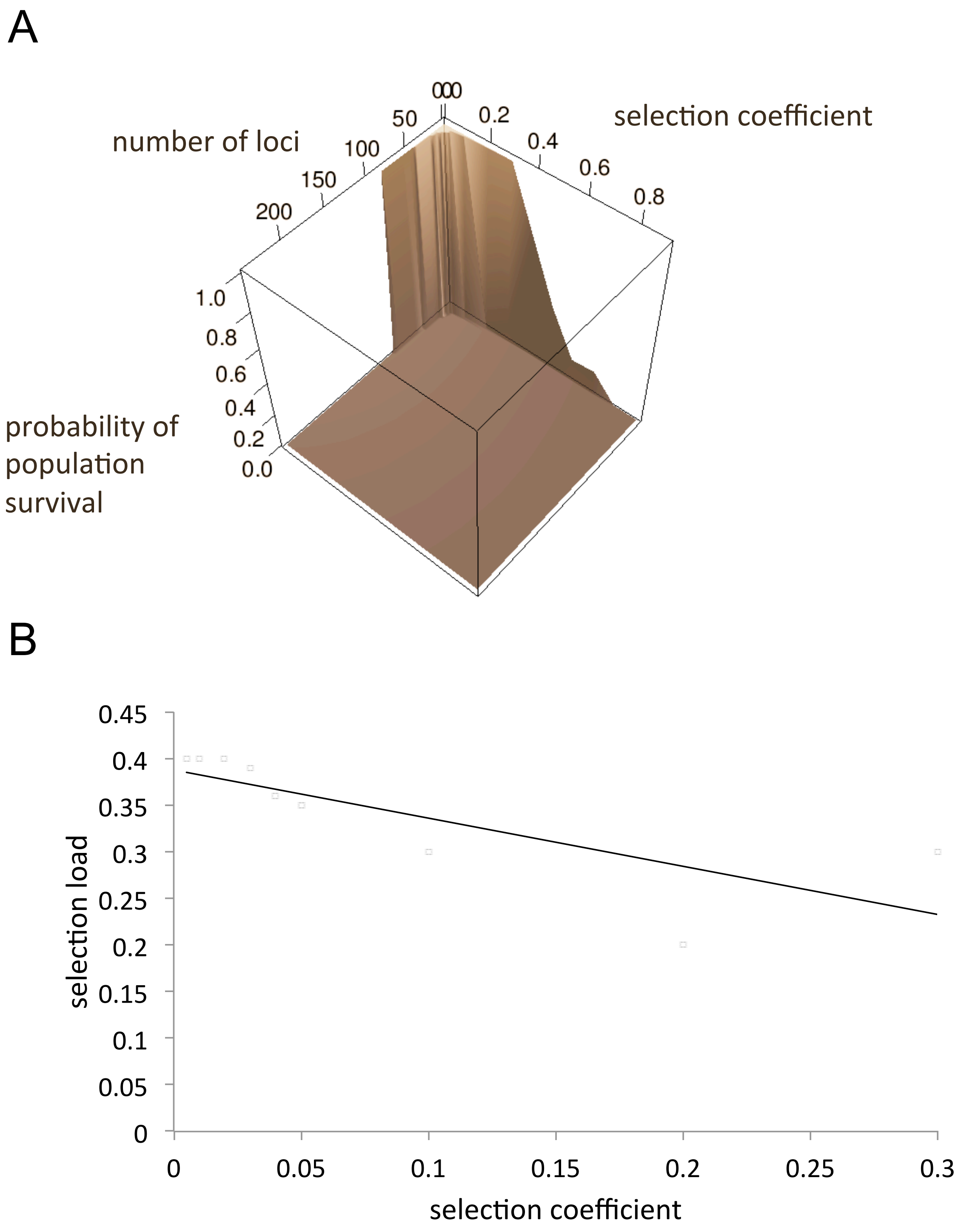
A
number of loci
000
50
100
150
200
0.2
0.4
0.6
0.8
selection coefficient
1.0
0.8
0.6
0.4
0.2
0.0
probability of population survival
B
selection load
0.45
0.4
0.35
0.3
0.25
0.2
0.15
0.1
0.05
0
0
0.05
0.1
0.15
0.2
0.25
0.3
selection coefficient

Figure S1.

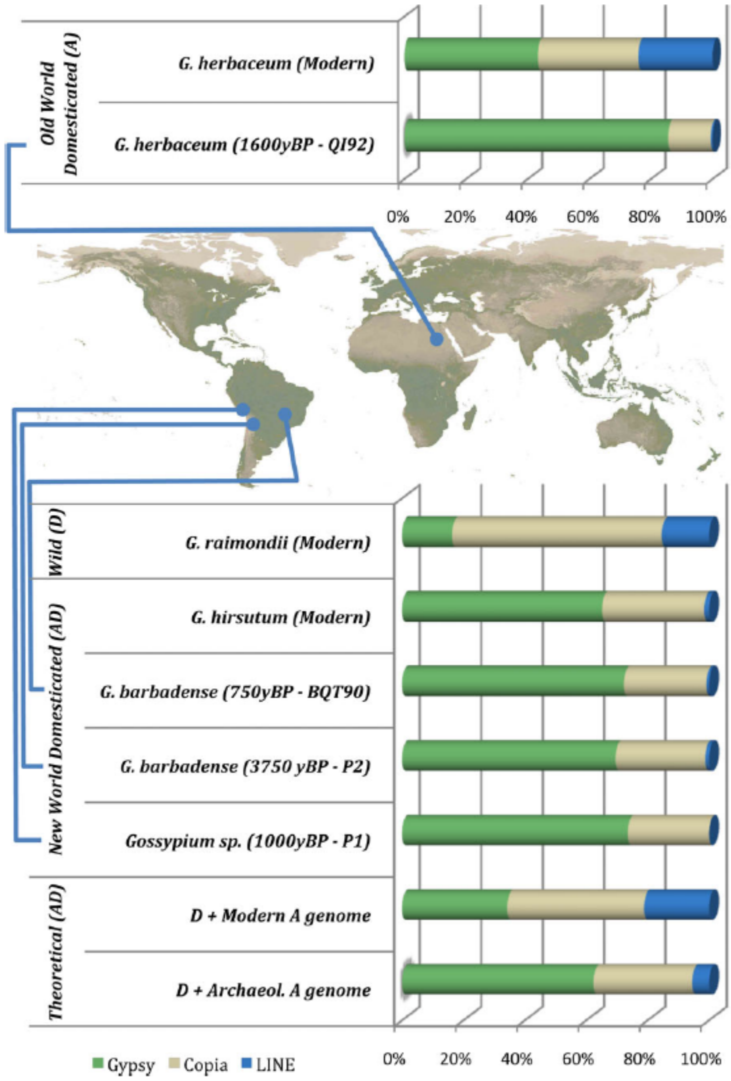
Old World Domesticated (A)
G. herbaceum (Modern)
G. herbaceum (1600yBP - QI92)
0%
20%
40%
60%
80%
100%
Wild (D)
G. raimondii (Modern)
New World Domesticated (AD)
G. hirsutum (Modern)
G. barbadense (750yBP - BQT90)
G. barbadense (3750 yBP - P2)
Gossypium sp. (1000yBP - P1)
Theoretical (AD)
D + Modern A genome
D + Archaeol. A genome
Gypsy
Copia
LINE
0%
20%
40%
60%
80%
100%

Figure S2.

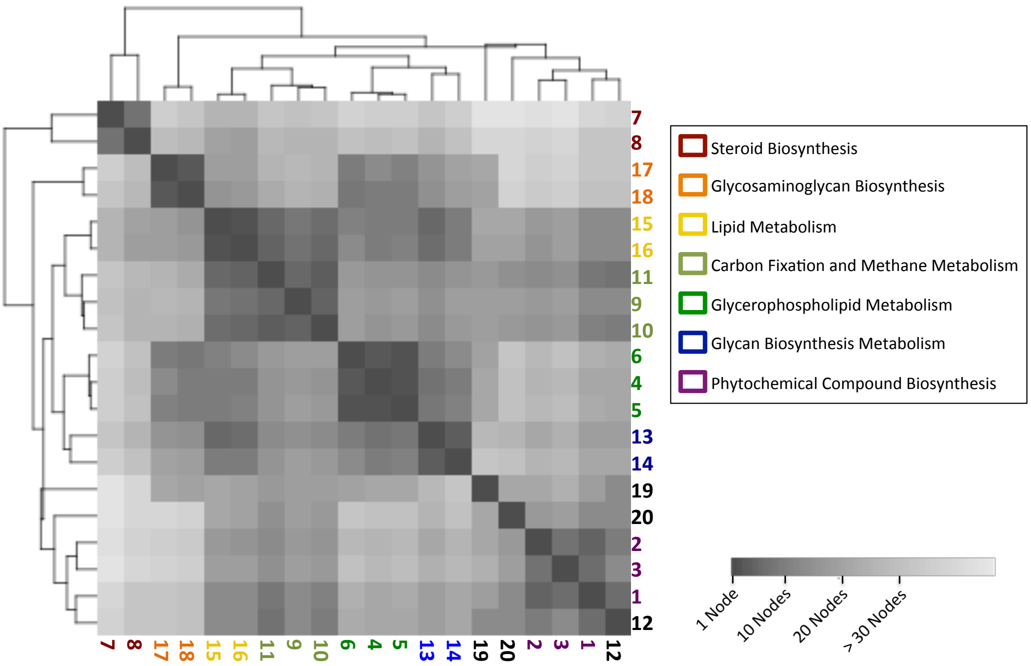
7
8
17
18
15
16
11
9
10
6
4
5
13
14
19
20
2
3
1
12
Steroid Biosynthesis
Glycosaminoglycan Biosynthesis
Lipid Metabolism
Carbon Fixation and Methane Metabolism
Glycerophospholipid Metabolism
Glycan Biosynthesis Metabolism
Phytochemical Compound Biosynthesis
1 Node
10 Nodes
20 Nodes
> 30 Nodes

Figure S3

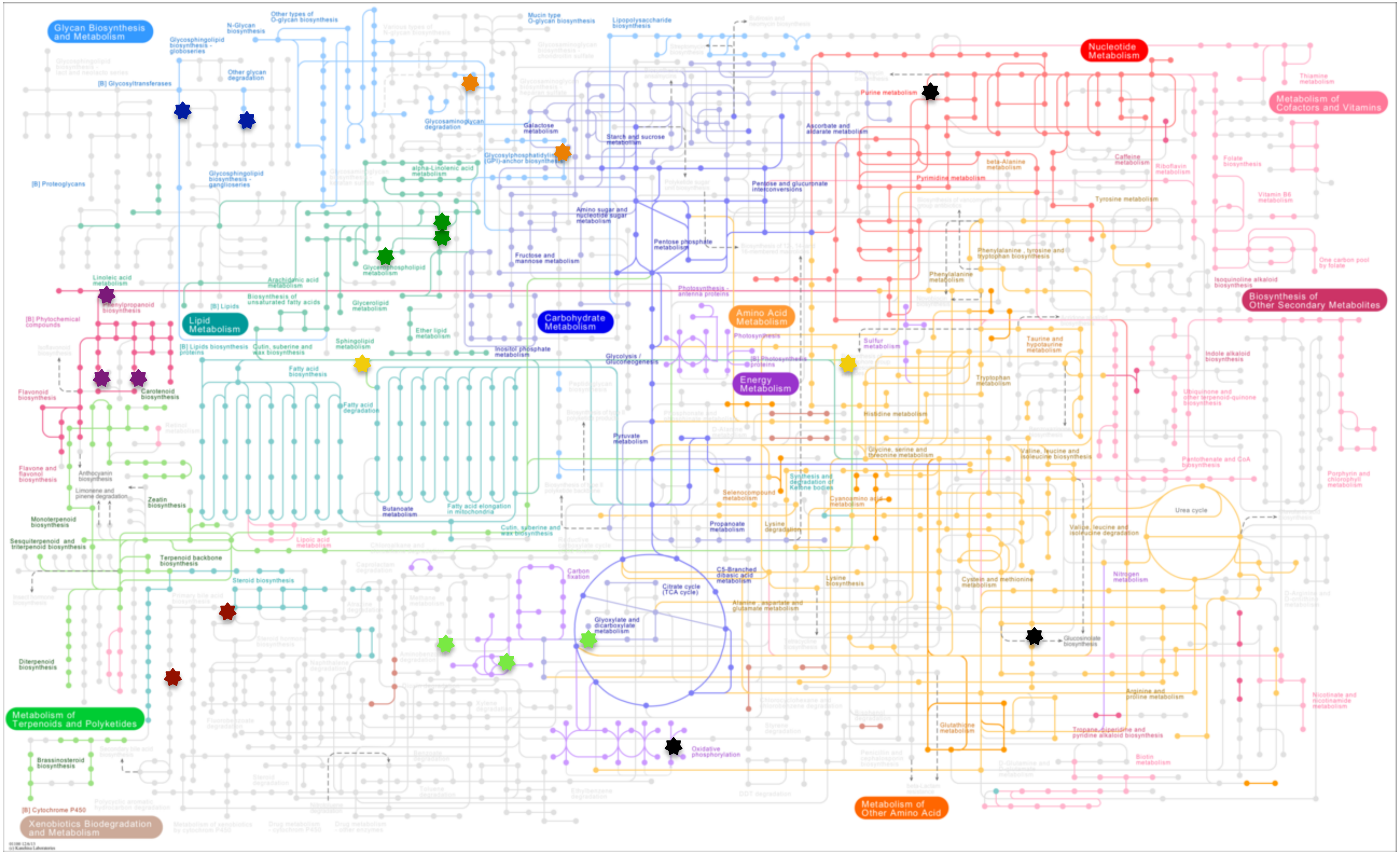

Glycan Biosynthesis and Metabolism
Lipid Metabolism
Carbohydrate Metabolism
Nucleotide Metabolism
Metabolism of Cofactors and Vitamins
Biosynthesis of Other Secondary Metabolites
Amino Acid Metabolism
Energy Metabolism
Metabolism of Terpenoids and Polyketides
Xenobiotics Biodegradation and Metabolism
Metabolism of Other Amino Acid
N-Glycan biosynthesis
Other types of O-glycan biosynthesis
Mucin type O-glycan biosynthesis
Lipopolysaccharide biosynthesis
Glycosphingolipid biosynthesis - globoseries
Other glycan degradation
[B] Glycosyltransferases
[B] Proteoglycans
Glycosphingolipid biosynthesis - ganglioseries
Glycosaminoglycan degradation
Galactose metabolism
Starch and sucrose metabolism
Glycosylphosphatidylinositol (GPI)-anchor biosynthesis
alpha-Linolenic acid metabolism
Amino sugar and nucleotide sugar metabolism
Pentose and glucuronate interconversions
Ascorbate and aldarate metabolism
Purine metabolism
Pyrimidine metabolism
beta-Alanine metabolism
Caffeine metabolism
Riboflavin metabolism
Folate biosynthesis
Thiamine metabolism
Vitamin B6 metabolism
Tyrosine metabolism
One carbon pool by folate
Isoquinoline alkaloid biosynthesis
Pentose phosphate metabolism
Fructose and mannose metabolism
Glycerophospholipid metabolism
Arachidonic acid metabolism
Linoleic acid metabolism
Phenylpropanoid biosynthesis
[B] Phytochemical compounds
[B] Lipids
Biosynthesis of unsaturated fatty acids
Glycerolipid metabolism
Ether lipid metabolism
Sphingolipid metabolism
[B] Lipids biosynthesis proteins
Cutin, suberine and wax biosynthesis
Inositol phosphate metabolism
Photosynthesis - antenna proteins
Photosynthesis
Phenylalanine, tyrosine and tryptophan biosynthesis
Phenylalanine metabolism
Taurine and hypotaurine metabolism
Sulfur metabolism
Indole alkaloid biosynthesis
[B] Photosynthesis proteins
Tryptophan metabolism
Ubiquinone and other terpenoid-quinone biosynthesis
Glycolysis / Gluconeogenesis
Fatty acid biosynthesis
Fatty acid degradation
Flavonoid biosynthesis
Carotenoid biosynthesis
Histidine metabolism
Pyruvate metabolism
Glycine, serine and threonine metabolism
Valine, leucine and isoleucine biosynthesis
Pantothenate and CoA biosynthesis
Porphyrin and chlorophyll metabolism
Flavone and flavonol biosynthesis
Anthocyanin biosynthesis
Limonene and pinene degradation
Zeatin biosynthesis
Synthesis and degradation of ketone bodies
Selenocompound metabolism
Cyanoamino acid metabolism
Butanoate metabolism
Fatty acid elongation in mitochondria
Monoterpenoid biosynthesis
Propanoate metabolism
Lysine degradation
Urea cycle
Valine, leucine and isoleucine degradation
Sesquiterpenoid and triterpenoid biosynthesis
Lipoic acid metabolism
Cutin, suberine and wax biosynthesis
Terpenoid backbone biosynthesis
Carbon fixation
C5-Branched dibasic acid metabolism
Citrate cycle (TCA cycle)
Lysine biosynthesis
Cystein and methionine metabolism
Nitrogen metabolism
Steroid biosynthesis
Alanine, aspartate and glutamate metabolism
Glyoxylate and dicarboxylate metabolism
Glucosinolate biosynthesis
Diterpenoid biosynthesis
Arginine and proline metabolism
Nicotinate and nicotinamide metabolism
Glutathione metabolism
Tropane, piperidine and pyridine alkaloid biosynthesis
Oxidative phosphorylation
Biotin metabolism
Brassinosteroid biosynthesis
[B] Cytochrome P450
